# A randomized, multi-center, open-label study to compare the safety and efficacy between afatinib monotherapy and combination therapy of afatinib and HAD-B1 for the locally advanced or metastatic NSCLC patients with EGFR mutations

**DOI:** 10.1097/MD.0000000000023455

**Published:** 2020-12-04

**Authors:** Si-Yeon Song, Su-Jeong Ha, Ji-Hye Park, So-Jung Park, Seong Hoon Shin, Chulho Oak, Jun-Yong Choi, Seong Woo Yoon, Jung-A Kim, Seong Hoon Yoon, Ji Woong Son, Seung Joon Kim, Hwa-Seung Yoo

**Affiliations:** a East West Cancer Center, Daejeon Korean Medicine Hospital of Daejeon University, Daejeon; b East West Cancer Center, Seoul Korean Medicine Hospital of Daejeon University, Seoul; c Department of Internal Medicine, Kosin University Gospel Hospital, Kosin University College of Medicine, Pusan; d Department of Internal Medicine, School of Korean Medicine & Korean Medicine Hospital of Pusan National University, Yangsan; e Korean Medicine Cancer Center, Kyung Hee University Hospital at Gangdong; f Division of Hematology-Oncology, Department of Internal Medicine, Kyung Hee University Gangdong Hospital, Seoul; g Department of Internal Medicine, Pusan National University Yangsan Hospital, Yangsan; h Department of Internal Medicine, Konyang University Hospital, Daejeon; i Department of Internal Medicine, Seoul St. Mary's Hospital, Postech-Catholic Biomedical Engineering Institute, College of Medicine, The Catholic University of Korea, Seoul, Republic of Korea.

**Keywords:** afatinib, HAD-B1, herbal medicine, non-small cell lung cancer, randomized controlled trial

## Abstract

**Background:**

Afatinib is an epidermal growth factor receptor - tyrosine kinase inhibitor (EGFR-TKI) with proven efficacy for treating patients with advanced or metastatic non-small cell lung cancer (NSCLC). Unfortunately, responses are limited by acquired resistance. Because traditional Korean medicine may have synergistic effects when combined with chemotherapy or radiotherapy, the aim of our study is to elucidate the efficacy and safety of afatinib plus HangAmDan-B1 (HAD-B1) combination therapy in the treatment of patients with NSCLC, as well as EGFR mutations, who need afatinib therapy.

**Methods/design:**

This study is a randomized, multi-center, open clinical trial. A total of 142 eligible subjects, recruited at 8 centers, are randomly assigned to take Afatinib (20–40 mg) ± HAD-B1 (0.972 g/day) for 16 weeks. In the test group, HAD-B1 and afatinib will be used in combination. The primary outcome is a comparison of starting dose maintenance rate as well as the disease control rate (DCR) between afatinib monotherapy and afatinib plus HAD-B1 combination therapy in patients with local advanced or metastatic (Stage IIIA, B, C/IV) NSCLC. Secondary outcomes are the Progression Free Survival (PFS), Time to progression (TTP), Overall survival rate, ORR based on RESIST 1.1, tumor size reduction, health-related quality of life (HRQoL), and Tumor marker.

**Discussion:**

The result of this clinical trial will provide evidence for the efficacy and safety of using HAD-B1 in the treatment of EGFR-positive patients with locally advanced or metastatic NSCLC who require afatinib therapy.

**Trial registration:**

Clinical Research Information Service (CRIS), Republic of Korea (ID: KCT0005414), on September 23, 2020.

## 1 Introduction

Around 85% of primary lung cancers are non-small cell lung cancer (NSCLC).^[1]^ Numerous oncogenic drivers, including mutations in the genes encoding the epidermal growth factor receptor (EGFR), have been recognized in NSCLC. EGFR mutations are found in 47% of lung adenocarcinomas, the most frequent NSCLC subtype in Asia-Pacific, and in 43% of the adenocarcinomas in the Republic of Korea.^[2]^ The activating mutations in EGFR lead to increased signaling downstream of the receptor, resulting in cellular growth and proliferation and in the promotion of tumor development due to enhanced metastatic spread and resisting apoptotic signals.^[3]^ Platinum-based chemotherapy has been the basis for the treatment of patients with advanced or metastatic NSCLC, but the availability and use of EFGR - tyrosine kinase inhibitors (EGFR TKIs) have significantly changed the management of patients with NSCLC harboring EGFR mutations. Gefitinib, erlotinib, and afatinib are 3 widely used EGFR-TKIs with proven efficacy for the treatment of patients advanced or metastatic NSCLC.^[4]^

In 2013, afatinib was approved by the U.S. FDA for the first line treatment of patients with locally advanced or metastatic NSCLC who harbor nonresistant EGFR mutations.^[5]^ The clinical effectiveness of afatinib in the treatment of such patients has been confirmed by the results of recent real-world studies.^[6]^ Despite the high disease control rates, almost all patients eventually experience acquired resistance-mediated disease progression. Therefore, new combination regimens are needed to delay or overcome this acquired TKI resistance.

HangAmDan-B1 (HAD-B1) is a traditional Korean medicine composed of *Panax ginseng C.A., Panax notoginseng Radix, Cordyceps militaris, and Boswellia carterii BIRDWOOD* (Table 1). Preclinical studies have shown that HAD-B1 has an anti-lung-cancer effect in xenograft animal model experiments using A549 lung cancer cells and A549/CR cells.^[7]^ The results revealed that the activities of caspase-3, -8, and -9 in the HAD-B1-treated group were generally increased in the same way as they were in the afatinib group, suggesting its potential as a novel therapeutic agent for use in afatinib plus HAD-B1 combined therapy for inhibiting acquired resistance. Also, our previous exploratory study to assess the efficacy and safety of HAD-B1 for dose-finding in EGFR-positive NSCLC subjects who need afatinib therapy is currently in progress.^[8]^

**Table 1:** Components of HAD-B1.

Scientific name	Plant parts used	Representative component	Amount (mg)
Panax Notoginseng (Burk) F.H. Chen	Root	Notoginsenoside R1	839
Cordyceps militaris	Fruit and corpus	Cordycepin	640
Panax ginseng C.A. Meyer	Root	Ginsenoside Rg1 and Rb1	640
Boswellia carterii BIRDWOOD	Mastic	a-boswellic acid, b-boswellic acid	400

The aim of this study is to use a multicenter, randomized, controlled trial to explore the efficacy and safety of combination therapy with afatinib plus HAD-B1 in the treatment of patients with locally advanced or metastatic NSCLC who have an activating EGFR mutation. Moreover a cold-heat pattern identification questionnaire (CHPIQ) of NSCLC patients will be conducted to explore whether the prognosis varies according to cold-heat pattern in Korean medicine.

## 2 Methods

### 2.1 Trial design

This clinical trial is a randomized, multi-centre, open-label study that aims to compare the safety and efficacy between afatinib monotherapy and combination therapy with afatinib plus HAD-B1 for the treatment of patients with locally advanced or metastatic EGFR-mutation-positive NSCLC. Participants will be randomized using a ratio of 1:1 into a treatment group (afatinib 20–40 mg/day plus HAD-B1 2 tablets [972 mg/day]) and a control group (afatinib 20–40 mg/day monotherapy) and will be treated for 16 weeks. As treatment continues, follow-up visits will be scheduled at Day 1, week 2, 4, 8, and 16. Physical examination, vital signs, ECOGPS, clinical laboratory examination, tumor marker examination, imaging diagnosis and tumor evaluation, health-related quality of life survey, combined drug confirmation, and abnormal case evaluation are all performed during each visit. Furthermore, for cold-heat pattern identification and genetic search studies, PMRA chip tests and cold-heat pattern identification surveys are performed prior to randomization.

### 2.2 Recruitment and consent

**Figure 1:**
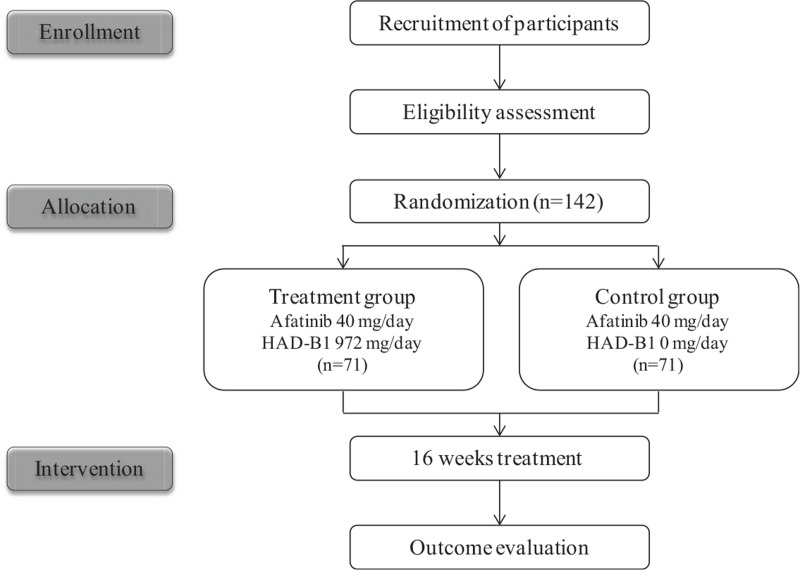
Study flow chart.

A total of 142 EGFR-mutation-positive patients with locally advanced or metastatic NSCLC patients who need afatinib therapy will be recruited at 8 centers. All subjects will undergo standardized interviews and receive clinical research information on the trial. Written informed consent will be obtained from each subject. The purpose, procedure, and potential benefits and risks of the study will be thoroughly explained to the subjects. The subjects can withdraw from the study at any time without consequence. The trial covers the period from October 31, 2020 to December 2, 2022 (anticipated), including the enrollment and follow-up periods (Fig. 1).

### 2.3 Inclusion criteria

Participants meeting the following criteria will be included:

1. Pathologically confirmed locally advanced or metastatic NSCLC (Stage IIIA-IV based on the eighth edition of the American Joint Commission on Cancer TNM staging system)^[9]^;
2. Mutated EGFR and requiring first line afatinib therapy (in the judgment of the researcher);
3. Disease measurable using Response Criteria for Clinical Trials of Cancer (RECIST) 1.1^[10]^;
4. ECOG performance status score from 0 to 2^[11]^;
5. Ability to take the drug orally (in the judgment of the researcher);
6. Nineteen years of age or older;
7. Willingness to sign a written consent to participate in this trial.

### 2.4 Exclusion criteria

Participants who meet 1 or more of the following criteria will be excluded:

1. Presence of a mutated T790M (threonine-to-methionine amino acid change at position 790) of the EGFR kinase domain (acquired, rebiopsy);
2. Presence of an active brain metastasis that has been stable for <4 weeks and with symptoms or leptomeningopathy; nevertheless, patients undergoing dexamethasone treatment may be allowed if a stable dose can be administered for at least 4 weeks;
3. Presence of gastrointestinal disorder with diarrhea, which is severe or unregulated within 2 weeks after screening, as a major symptom; for example, Crohn disease, absorption disorder, or pathological diarrhea with common terminology criteria (CTC) grade 2 or higher (by assessment of the researcher);
4. Presence of interstitial lung disease assessed as unsuitable for this trial by the researcher;
5. Severe hepatopathy (Child Pugh C);
6. Severe nephropathy (eGFR <15 ml/minute) or the need for dialysis;
7. Clinically significant cardiovascular disease (New York Heart Association (NYHA) class 3 congestive heart failure, unstable angina or uncontrolled arrhythmia, myocardial infarction, angina within 1 year of study participation, etc.);
8. Fertile women who do not use proper contraception prior to the start of this clinical trial;
9. Lactating women or pregnant women;
10. Suspicion of having or having been diagnosed with serious mental illness, such as drug abuse, alcoholism, etc;
11. Hypersensitivity to afatinib or other EGFR medications;
12. Hypersensitivity to HAD-B1 or its constituents;
13. Participation in any other clinical trials within the previous month before the;
14. Unsuitability for this clinical trial, as determined by the researcher, including severe infectious disease or organ failure.

### 2.5 Interventions

Patients in the treatment group will receive of 1 capsule/day of afatinib (20–40 mg/day orally) plus 2 HAD-B1 tablets (972 mg/day, bid orally) while those in the control group will only receive 1 capsule/day of afatinib (20–40 mg/day orally), which may be changed based on the medical judgment of the investigator. The investigator may extend the duration of the drug’s administration. The HAD-B1 tablets used in this trial will be purchased from the Hanpoong Pharmaceutical Co., Ltd. (Seoul, Korea). HAD-B1 is a medical substance containing *Panax ginseng C.A.* (640 mg), *Panax notoginseng Radix* (839 mg), *Cordyceps militaris* (640 mg), and *Boswellia carterii BIRDWOOD* (480 mg) as raw material medicines. In the toxicity assessment of HAD-B1 orally administered to rats for 13 consecutive weeks, the no-observed-adverse-effect-level (NOAEL) for HAD-B1 was determined as 2000 mg/kg/day. The human equivalent dose (HED) was calculated as approximately 320 mg/kg/day, which was about 19 g/day for a person of 60 kg.^[12]^ The dose of HAD-B1 in this clinical trial is approximately 1/20 of that. In the treatment group, when afatinib is removed, HAD-B1 is also removed; however, when afatinib is reduced, HAD-B1 is not reduced.

### 2.6 Outcome measures

**The primary measurement** is comparison of starting dose maintenance rate for afatinib and disease adjustment rate (DCR) at 16 weeks in accordance with RECIST 1.1 of Afatinib + HAD-B1 combined group and afatinib monotherapy group. The secondary outcomes are comparisons of the Progressive Free Survival (PFS) determined by RESIST 1.1, overall survival rate (OS), objective response rate (ORR), tumor size reduction, time to progression (TTP), HRQoL, and tumor markers (CEA, NSE) between the 2 groups. The PFS, ORR, DCR, and TTP are evaluated on the basis of RECIST 1.1 criteria.^[10]^ This clinical trial will explore whether the prognosis varies according to cold-heat pattern in Korean medicine. Cold-heat pattern identification will be evaluated using the usual symptom-based CHPIQ, which has been recognized for its reliability and validity.^[13]^

### 2.7 Safety assessments

During the study period, subjects will be continuously monitored for frequency, severity, and aspect of events of special interests (ESIs). ESIs include paronychia, loss of appetite, diarrhea, stomatitis, cheilitis, rash, acne dermatitis, interstitial lung disease, and severe hepatopathy. The overall safety profile will be evaluated by using safety indicators such as the numbers of abnormal cases and adverse drug reactions, the frequency of significant abnormalities, as well as severities and aspects of abnormalities, physical examination findings, abnormal vital signs, clinical laboratory findings, and clinically significant changes in the ECOG PS scores. The treatment discontinuation rate and reason will be recorded.

In this clinical trial, the “Data and Safety Monitoring Board (DSMB)” will be operated as a safety protection measure for the test subjects. The board will meet every 6 months after the commencement of the study to evaluate the overall safety of clinical trials.

### 2.8 Randomization and blinding

Random assignment will be performed using a computer-generated, blocked, random-allocation sequence with a 1:1 ratio. Each participant will be assigned to a group according to a pre-generated randomization table and an assigned randomized number. Each participant will be assigned to a treatment group, or the control group according to the allocation codes of the randomized assignment method, which will be prepared in advance. In this clinical trial, blinding is not performed.

### 2.9 Sample size calculation

According to the paper, the Afatinib group’s starting dose maintenance rate is assumed to be 57.6 percent, while the Afatinib + HAD B1 group’s starting dose maintenance rate is assumed to be 80.0 percent. Furthermore, it is assumed that all patients will be recruited and monitored for at least 16 weeks. As a result, each group has 64 test subjects. With a 10% dropout rate, 71 people will be recruited for a total of 142 people.^[14]^

### 2.10 Statistical analysis

#### 2.10.1 Analysis of first outcome variables

The frequency and percentage of afatinib’s initial dose maintenance rate for afatinib and disease control rate (DCR) according to RECIST 1.1 up to 16 weeks relative to baseline will be presented, and the statistical significance of differences between groups will be analyzed using Chi-square test or Fisher’s effect test.

#### 2.10.2 Analysis of second outcome variables

We will compare and evaluate the following between the treatment group and the control group at each time point from the baseline (0 week) to 16 weeks: Progressive survival period (PFS), the time to progression (TTP), and the overall survival rate (OS) present descriptive statistics (number of subjects tested, mean, standard deviation, median, minimum and maximum) and compare statistical significance through Kaplan–Meier analysis. The frequency and percentage of changes in objective response rates (ORR), QOL (EORTC QLQ-C30, EORTC QLQ-LC13, EQ-5D) according to RECIST 1.1 will be presented, and the statistical significance of differences between groups will be analyzed using Chi-square test or Fisher’s exact test.

The reduction of tumor size and tumor markers present descriptive statistics by dose group (number of subjects, mean, standard deviation, median, minimum, and maximum), and statistical significance of differences between groups is to be analyzed by two-sample-test or Wilcoxon signified rank test.

The statistical significance of the variation within each group is tested as either a paired *t*-test or a Wilcoxon signed rank test.

#### 2.10.3 Exploratory outcome variables

We will explore whether treatment reaction and prognosis vary according to cold-heat pattern predisposition. For categorical variables, the frequency and the percentage for each administration group will be presented, and the statistical significance for the differences between groups will be tested by using the Chi-Squared test or Fisher exact test. For continuous variables, descriptive statistics (number of subjects, mean, standard deviation, median value, minimum, and maximum values) for each group will be presented, and the statistical significance of differences between groups will be tested by using the two-sample *t* test or Wilcoxon signed rank test.

### 2.11 Safety assessment

The number of subjects who will present with adverse events, adverse drug reactions, serious adverse events, and events of special interests, as well as the ratio of those subjects to the total number in the group at baseline, will be recorded. The significance of differences between groups will be tested by using the Chi-Squared test or Fisher exact test. The amount of change at 16 weeks of treatment compared to the baseline for laboratory tests, vital signs, and electrocardiogram (ECG) results will be compared between groups. For continuous variables, descriptive statistics (number of subjects, mean, standard deviation, median value, minimum and maximum values) for the amount of change at 16 weeks of treatment compared to the baseline will be presented, and the statistical significance of differences between groups will be tested by using the paired *t* test or the Wilcoxon signed rank test. While for categorical variables among laboratory tests and ECG results, their frequency will be presented using a contingency table for normal/abnormal changes at 16 weeks of treatment compared to the baseline, and the significance of changes between groups will be tested by using the *t* test, Chi-Squared test, or Fisher exact test.

### 2.12 Data management

Clinical trial data will be collected by electronic data capture (EDC) system. Subject data will be entered into the electronic Case Report Form (e-CRF) according to the e-CRF instructions provided by the sponsor. The sponsor or its agent will perform verification of the supporting documents. After verifying the data, missing, or inconsistent data should be resolved using the EDC system. The investigator in charge shall digitally sign the locked e-CRF after verification of the data. Records of the subjects identity will be kept confidential. However, sponsors, IRB committees, monitors, and directors of the Korean Food and Drug Administration (KFDA) involved in this trial may view the subjects records for the purpose of monitoring, managing and reviewing the progress of the study. All data should be kept confidential and in place with confidentiality facilities and management standards. All documents related to the study, such as electronic case records, shall be recorded and discriminated by subject identification code, not subjects name.

## 3 Discussion

Afatinib is an ErbB family of blockers that inhibit the EGFR signaling pathway by irreversibly fastening to the ATP domain of ErbB family kinase domains. The efficacy and safety of afatinib in the management of patients with advanced-stage NSCLC harboring EGFR mutations were evaluated in a comprehensive clinical trial program (LUX-Lung) that included several, randomized studies of afatinib as a first-, second-, or third-line treatment for patients with lung adenocarcinomas.^[15–21]^ Despite initial positive responses to EGFR-TKIs, an acquired resistance inevitably leads to disease progression in most patients within 9 to 13 months, and major adverse events grade 3 or higher, including diarrhea, rash or acne, and stomatitis or mucositis, occurred.^[21–23]^

Interest in complementary and alternative medicines for the treatment of patients with cancer because of their synergistic effects when combined with western medicine is growing in Korea. HAD-B1 has shown a potential synergetic therapeutic effect in conjunction with western medicine in vitro or in vivo studies.^[24–27]^ One multicenter, randomized, controlled trial reported that EGFR-TKI with Chinese herbal medicine improved the PFS (13.50 months; 95% CI, 11.20–16.46 months) when compared with TKI alone (10.94 months; 95% CI, 8.97–12.45 months; hazard ratio, 0.68; 95% CI, 0.51–0.90; *P* = .0064) in patients with EGFR mutation-positive NSCLC plus less common adverse events.^[28]^ The extended PFS and the overall survival (OS) in cancer patients using traditional medicine in conjunction with EGFR-TKIs have been demonstrated in various clinical studies.^[28–30]^ However, those studies had either been retrospective or had small samples; moreover, the influence of the cold-heat pattern was never studied. The cold-heat pattern is 1 diagnostic method frequently used in Korean medicine. In this clinical trial, in order to find a customized precision medicine system for the treatment of patients with lung cancer, we will identify the cold-heat patterns of the patients by using an objective and reliable cold-heat questionnaire to determine whether the prognosis varies depending on the cold-heat pattern.

In light of the above, this prospective, multicenter, randomized, controlled trial will verify the efficacy and safety of a combination therapy of afatinib plus HAD-B1 in treating patients with locally advanced or metastatic NSCLC. At the same time, this trial may provide clinical evidence supporting integrative medicine and may propose synergistic effects of treating such patients with this combination therapy.

## Author contributions

**Conceptualization:** Su-Jeong Ha, Si-Yeon Song, Seong Hoon Shin.

**Funding acquisition:** Hwa-Seung Yoo.

**Writing – original draft:** Su-Jeong Ha, Si-Yeon Song, Ji-Hye Park, So-Jung Park, Seong Hoon Shin, Chulho Oak, Jun-Yong Choi, Seong Woo Yoon, Jung-A Kim, Seong Hoon Yoon, Ji Woong Son, Seung Joon Kim, Hwa-Seung Yoo.

**Writing – review & editing:** Su-Jeong Ha, Si-Yeon Song, Ji-Hye Park, So-Jung Park, Seong Hoon Shin, Chulho Oak, Jun-Yong Choi, Seong Woo Yoon, Jung-A Kim, Seong Hoon Yoon, Ji Woong Son, Seung Joon Kim, Hwa-Seung Yoo.
