# A randomized, multi-center, open-label study to compare the safety and efficacy between afatinib monotherapy and combination therapy of afatinib and HAD-B1 for the locally advanced or metastatic NSCLC patients with EGFR mutations: Erratum

**DOI:** 10.1097/MD.0000000000028585

**Published:** 2022-01-14

In the protocol, “Multi-center, Randomized, Double-blind, Placebo-controlled, Exploratory Study to Evaluate the Efficacy and Safety of HAD-B1 for Dose-finding in EGFR positive and locally advanced or metastatic NSCLC subjects who need Afatinib therapy”,^[1]^ which appeared in Volume 99, Issue 49 of *Medicine*, the authors would like to note the following updates to the protocol. Updates were made in response to the recently announced results that Afatinib's mPFS was deemed insufficient for 48 weeks of observation, which makes obtaining Afatinib's anticancer care benefits for the original clinical trial (48 weeks) difficult.^[2]^ The primary endpoint, secondary outcome, trial design study period, sample size calculation, follow-up visits, heath-related quality of life evaluation, outcome measurement, and statistical analysis of first and second outcome variables were updated to reflect the announced results. The changes to the original article include:

- Throughout the article
  ∘ The total number of eligible subjects changes from 178 to 142.
  ∘ The number of weeks subjects took Afatinib changes from 48 to 16.
- In the abstract:
  ∘ “The primary outcome is ac comparison of progression free survival (PFS)…” changes to “Starting dose maintenance rate as well as the disease control rate (DCR)…”
  ∘ “Secondary outcomes are the overall survival rates, clinical responses, tumor size reductions, health-related qualities of life, and safety” changes to “Secondary outcomes are Progression Free Survival (PFS), Time to progression (TTP), Overall survival rate, ORR based on RESIST 1.1, tumor size reduction, health-related quality of life (HRQoL), and Tumor marker.”
- Page 2
  ∘ “…follow-up visits will be scheduled at 2, 4, 8, 16, 24, 32, 40 and 48 weeks” has been updated to “follow-up visits will be scheduled at day 1, week 2, 4, 8, and 16.”
  ∘ “At each visit, vital signs will be taken, physical and clinical laboratory examinations will be performed, as will tumor marker tests, imaging diagnoses, and tumor response evaluations. Scores on the Eastern Cooperative Oncology Group Performance Status scale (ECOG PS) will be recorded, and a health-related quality of life (HRQoL) questionnaire survey, combined drug identifications, and evaluations of abnormal cases will be conducted. In addition, CHPIQ will be administered before randomization. After the progression of the disease or after 48 weeks, the survival of the subjects will be surveyed at intervals of 8 to 12 weeks, their tumor responses on the testers judgment will be evaluated” has been updated to “Physical examination, vital signs, ECOGPS, clinical laboratory examination, tumor marker examination, imaging diagnosis and tumor evaluation, health-related quality of life survey, combined drug confirmation, and abnormal case evaluation are all performed during each visit. Furthermore, for cold-heat pattern identification and genetic search studies, PMRA chip tests and cold-heat pattern identification surveys are performed prior to randomization.”
- Figure 1
  ∘ “Randomization (n = 178)” has been corrected to “Randomization (n = 142).”
  ∘ “n = 89” has been corrected to “n = 71”.
- Page 3
  ∘ “Mutated EGFR and requiring afatinib therapy (in the judgment of the researcher)” has been corrected to “Mutated EGFR and requiring first line afatinib therapy (in the judgment of the researcher)”.
  ∘ “Afatinib and HAD-B1 will be administered continuously for 48 weeks” has been corrected to “The investigator may extend the duration of the drug's administration.”
- Page 4
  ∘ “The primary outcome of this trial is a comparison of the progression-free survival (PFS) between the afatinib monotherapy and combination therapy of afatinib plus HAD-B1 for patients with locally advanced or metastatic EGFR-mutationpositive NSCLC” has been corrected to “**The primary measurement** is comparison of starting dose maintenance rate for afatinib and disease adjustment rate (DCR) at 16 weeks in accordance with RECIST 1.1 of Afatinib + HAD-B1 combined group and afatinib monotherapy group.”
  ∘ “disease control rate (DCR)” has been removed from the second sentence of section 2.6.
  ∘ Section 2.9 has been updated from “The sample size was determined taking into account the number of subjects available, the minimum range of the efficacy assessment, and the expected dropout rate during the study eriod. When the first 2 factors were considered, the total number of subjects required for each group was calculated as 80, for a total of 160.When a 10% dropout rate was applied to each group, the number of subjects required was calculated as 89 (80/(1–0.1) = 88.89 ≒ 89). Therefore, considering all factors, the number of subjects to be registered for each group is 89, so a total of 178 patients will be recruited” to “According to the paper, the Afatinib group's starting dose maintenance rate is assumed to be 57.6 percent, while the Afatinib + HAD B1 group's starting dose maintenance rate is assumed to be 80.0 percent. Furthermore, it is assumed that all patients will be recruited and monitored for at least 16 weeks. As a result, each group has 64 test subjects. With a 10% dropout rate, 71 people will be recruited for a total of 142 people. [30]”
  ∘ Section 2.10.1 has been updated from “Descriptive statistics (number in each group, mean, standard deviation, median value, minimum value and maximum value) will be presented to compare the progression free survival (PFS) for the treatment group to that for the control group for up to 48 weeks and to compare each group to its baseline (week 0) data. The difference between the treatment group and the control group will be analyzed using a Kaplan–Meier survival analysis” to “The frequency and percentage of afatinib's initial dose maintenance rate for afatinib and disease control rate (DCR) according to RECIST 1.1 up to 16 weeks relative to baseline will be presented, and the statistical significance of differences between groups will be analyzed using Chi-square test or Fisher's effect test.”
  ∘ Section 2.10.2 has been updated from “We will compare and evaluate the following between the treatment group and the control group at each time point from the baseline (0 week) to 48 weeks: The statistical significance of OSs and TTPs will be compared and analyzed through Kaplan–Meier analyses. The frequency and the percentage of changes in ORRs, DCRs, QOLs (EORTC QLQ-C30, EQ-5D) according to RECIST 1.1 will be presented for each administration group, and the statistical significance of the differences between groups will be analyzed by using the Chi-Squared test or Fisher exact test. Descriptive statistics (number of subjects, mean, standard deviation, median value, minimum and maximum values) will be presented for reductions in tumor size and tumor markers for each administration groups, and the statistical significance of the differences between groups will be analyzed by using the twosample t test or the Wilcoxon signed rank test. The statistical significance of the amount of change within each group will be tested using the paired t test or the Wilcoxon signed rank test” to “We will compare and evaluate the following between the treatment group and the control group at each time point from the baseline (0 week) to 16 weeks: Progressive survival period (PFS), the time to progression (TTP), and the overall survival rate (OS) present descriptive statistics (number of subjects tested, mean, standard deviation, median, minimum and maximum) and compare statistical significance through Kaplan Meier analysis.The frequency and percentage of changes in objective response rates (ORR), QOL (EORTC QLQ-C30, EORTC QLQ-LC13, EQ-5D) according to RECIST 1.1 will be presented, and the statistical significance of differences between groups will be analyzed using Chi-square test or Fisher's exact test. The reduction of tumor size and tumor markers present descriptive statistics by dose group (number of subjects, mean, standard deviation, median, minimum, and maximum), and statistical significance of differences between groups is to be analyzed by two-sample-test or Wilcoxon signified rank test. The statistical significance of the variation within each group is tested as either a paired t-test or a Wilcoxon signed rank test.”
